# Validation of Automated Somatotype Estimation Proposal Using Full-Body 3D Scanning

**DOI:** 10.3390/bioengineering12070717

**Published:** 2025-06-30

**Authors:** Bibiána Ondrejová, Lucia Bednarčíková, Norbert Ferenčík, Jozef Živčák

**Affiliations:** Department of Biomedical Engineering and Measurement, Faculty of Mechanical Engineering, Technical University of Košice, 042 00 Košice, Slovakia; lucia.bednarcikova@tuke.sk (L.B.); norbert.ferencik@tuke.sk (N.F.)

**Keywords:** 3D body scanning, somatotype, anthropometric analysis, body composition

## Abstract

Somatotyping is essential for assessing body composition in sports science, anthropology, and medicine. Traditional methods, such as the Heath–Carter approach, rely on manual measurements, which can be prone to errors and variability. This study evaluates the validity and reliability of 3D body scanning as an alternative to manual somatotyping. A total of 117 participants (49 males, 68 females) aged 18 to 27 years were assessed using both traditional anthropometric methods and a full-body 3D scanning system (TC^2^ NX-16). The three somatotype components (ectomorphy, mesomorphy, and endomorphy) were calculated using the Heath–Carter method. A custom-developed application processed the scanned data to compute somatotype values. The results were compared using statistical metrics, including intraclass correlation coefficients (ICCs) and Bland–Altman analysis. The 3D scanning method showed high agreement (87.18%) with manual measurements. Minor discrepancies were observed particularly in the endomorphic component, which was slightly overestimated by 3D scanning. Mesomorphic and ectomorphic components exhibited minimal differences. Statistical analyses confirmed strong reliability with ICC values exceeding 0.87. Conclusions: Full-body 3D scanning is a viable, non-invasive, and efficient alternative to traditional somatotyping methods. Despite minor differences in endomorphy estimation, the overall accuracy and reliability supports its use in sports science, health monitoring, and anthropometric research. Future studies should refine predictive models for endomorphy estimation and integrate AI-driven classification techniques to enhance precision.

## 1. Introduction

Somatotyping is a crucial method for assessing body composition and structure that is widely applied in anthropology, sports science, and medicine. Traditional methods, such as the Heath–Carter approach, rely on manual measurements of body parameters, which can lead to subjective errors and are time consuming. The advancement of modern technologies, particularly 3D scanning, offers an opportunity to automate this process, enhancing measurement accuracy and reliability [1,2,3].

The primary motivation for this research is the need to improve and streamline somatotype determination without relying on manual measurements. Traditional methods require trained professionals and can exhibit significant inter- and intra-individual variability. The 3D body scanners enable the acquisition of extensive anthropometric data with high precision while minimizing human error [3,4].

Recent research has shown that 3D scanning technology can be applied in somatotyping, offering a non-invasive and rapid method for capturing body dimensions. Several studies have explored the integration of 3D body scans with traditional anthropometric classification systems. However, certain limitations remain. Optical 3D scanners often struggle to accurately capture the top of the head due to the presence of hair or facial hair, which can result in inconsistent height measurements and subsequently affect ectomorphy calculation. Moreover, estimating endomorphy typically relies on skinfold measurements, which still require direct physical contact and are highly dependent on the evaluator’s experience [2,3,4,5,6].

Automated somatotype assessment using 3D scanning has the potential to innovate anthropometric analyses in sports science, medicine, and body composition research. This approach allows for fast, accurate, and standardized body evaluations, which can contribute to better diagnostics, personalized training programs, and more efficient monitoring of physical changes during exercise or health interventions [6,7,8,9,10].

This study aims to enhance and automate somatotype determination using the full-body 3D scanner by eliminating the need for manual measurements and improving the efficiency of the process. By developing a fully automated somatotyping system, this research has the potential to transform anthropometric analysis, making it more efficient, accurate, and accessible for applications in sports science, medicine, and body composition research.

## 2. Materials and Methods

The study received institutional ethical approval before commencing. All tests were conducted in the labs at the university campus with a comfortable temperature. Volunteers over 18 years old were invited to participate in this study. All data used in this study were fully anonymized and processed solely under unique identification codes; therefore, informed consent was not required.

### 2.1. Participants

A total of 117 subjects (49 males, 68 females) aged 18 to 27 years participated with a mean age of 22.1 ± 4.63 years, height of 171 ± 8.98 cm, and weight of 69.86 ± 14.49 kg. Each participant was measured using traditional manual anthropometric techniques and had 3D scan data captured using a full-body 3D scanning system TC^2^ NX-16 ([TC]^2^ Labs, Raleigh, NC, USA).

### 2.2. Traditional Anthropometric Method

Anthropometric data collected from participants included: height, weight, skinfolds of triceps, subscapular, supraspinale, medial calf, girths of bicep and calf as well as bone breadths of humerus and femur. The traditional anthropometric method involved manual measurements using skinfold calipers SH5020 (Saehan Corporation, Masanhappo-gu, Changwon, Republic of Korea), measuring tape SH5023 (Saehan Corporation, Masanhappo-gu, Changwon, Republic of Korea), and bone calipers (HMG Direct, Brisbane, QLD, Australia). All manual anthropometric measurements were collected in accordance with the International Standards for Anthropometric Assessment (ISAK) protocol, which defines standardized procedures for measuring skinfolds, girths, and bone breadths [11]. Each anthropometric parameter was recorded three times by the same trained observer, and the mean of the three values was used for subsequent analysis. These data were used to calculate the three somatotype components (ectomorphy, mesomorphy, and endomorphy) from manually measured values using the standard equations of the Heath–Carter method. These measurements were extracted to Excel (Microsoft, Redmond, WA, USA), and calculations were performed in developed PC applications and served as a reference for model development and evaluation.

### 2.3. 3D Body Scanning

The TC^2^ NX-16 full-body 3D scanning system ([TC]^2^ Labs, USA) was used to acquire precise anthropometric data (point accuracy of <1 mm and circumference measurement accuracy of <3 mm, as specified by the manufacturer). This system employs a multi-camera setup with structured light scanning technology to generate high-resolution three-dimensional body measurements within eight seconds. The scanner captures circumferences, bone breadths, and body dimensions in a non-invasive and standardized manner, ensuring consistency and minimizing measurement errors.

All participants were instructed to wear light-colored, form-fitting, non-compressive undergarments with female participants additionally required to wear a snug, non-compressive sports top during data collection. Participants stood in a designated scanning position with arms slightly away from the body to allow for accurate limb measurements. The scanning process was completed within seconds, producing a full-body digital model that included key anthropometric parameters such as limb circumferences (upper arm, calf) and bone breadths (humerus, femur). Anatomical landmarks and anthropometric features were automatically detected based on curvature analysis and body surface segmentation using the scanner’s proprietary algorithm. No manual landmark placement or post-processing adjustment was applied. Each scan was verified for completeness and posture alignment before inclusion in the dataset.

The extracted 3D body measurements were then processed using a prototype PC-based application, which automatically computed somatotype parameters based on the Heath–Carter method. These automated calculations were later compared to manual measurements for model validation and performance assessment.

### 2.4. PC Application Development

The PC application, developed in C# using the Microsoft .NET Framework (Microsoft Corporation, USA), is designed for both manual data entry and automatic data import from the TC^2^ NX-16 ([TC]^2^ Labs, USA) full-body 3D scanner. To develop the application, we used the Visual Studio 2022 (Microsoft Corporation, Redmond, WD, USA) environment in the Windows 11 Pro (Microsoft Corporation, Redmond, WD, USA) operating system.

**Figure 1:**
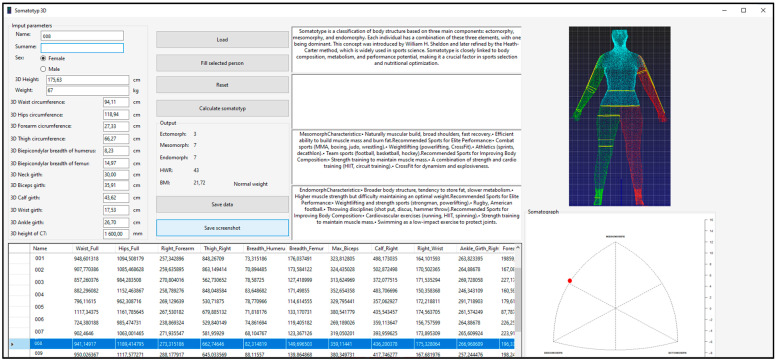
The interface of the developed computer application for somatotype calculation was designed to process data from manually measured inputs and imported data from a 3D scanner.

The front-end interface of the developed application features a control GroupBox, which facilitates the loading of an entire Excel dataset into a tabular format within the program via a designated button, as illustrated in Figure 1. Upon selecting a specific row corresponding to the evaluated individual, the relevant parameters are loaded, and all text fields are automatically populated with the respective data except for the subject’s weight, which must be manually entered. Subsequently, by pressing the “Calculate Somatotype” button, the application automatically computes the individual’s ectomorph, mesomorph, and endomorph values, as well as the height–weight ratio (*HWR*), body mass index (*BMI*), and a BMI-based assessment parameter. Simultaneously, the system generates a somatograph visualization along with additional text fields relevant to the selected somatotype representation. The computed parameters can be saved to a user-specified location for further analysis. All calculated values, i.e., ectomorph, mesomorph, and endomorph as well as height–weight ratio (*HWR*), are rounded to a whole number, while the *BMI* index contains two decimal places.

In addition to its core functionality, the application supports data import and export in Excel format, ensuring seamless integration with statistical and analytical tools. The program enables users to determine somatotype components based on the Heath–Carter method, using either manually recorded measurements or scanner-derived anthropometric data. The application is designed for execution on Windows operating systems from Windows XP onwards provided the necessary framework is available. For data import and export operations, Microsoft Excel 2007 or later is required due to table structure compatibility. To enhance data integrity, the application incorporates validation mechanisms to prevent errors caused by incorrect table dimensions. Notably, the software is entirely self-contained, and it was developed without reliance on external libraries.

### 2.5. Estimation Model Development from 3D Scan

Since the 3D scanner does not capture head height due to hair occlusion and skinfolds thickness, height and body fat estimation were integrated into the ectomorphy and endomorphy calculation using an alternative predictive approach.

#### 2.5.1. Endomorphy Calculation

The endomorphic component, which represents relative fatness, was estimated using the US Navy body fat equation (metric version) [12] as a replacement for skinfold measurements. The calculated body fat percentage (*BF*%) was then converted to an endomorphy value using the assumption that 5% body fat corresponds to 1 unit of endomorphy [13].

For males [12]:

*BF* (%) = 495/(1.0324 − 0.19077 ∗ log (waist − neck) + 0.15456 ∗ log (height)) − 450, (1)

For females [12]:

*BF* (%) = 495/(1.29679 − 0.35004 ∗ log (waist + hips − neck) + 0.22100 ∗ log (height)) − 450, (2)

where all variables are measured in centimeters (cm); *BF* (%) is body fat percentage. Logarithms are base 10.

Endomorphy conversion:

Endomorphy = *BF* (%)/5, (3)

#### 2.5.2. Ectomorphy Calculation

The ectomorphic component, which represents linearity and leanness, is traditionally determined by the height-to-weight ratio (*HWR*):

(4) $$HWR=\frac{Height}{{Weight}^{1/3}}$$

where height is in centimeters (cm) and weight is in kilograms (kg). The *HWR* (height-to-weight ratio) is a dimensionless index used in the Heath–Carter method for classifying ectomorphy.

The calculated *HWR* was then used to classify ectomorphy as follows:

1. If *HWR* ≥ 40.75 → Ectomorphy = 0.732 × *HWR* − 28.58.
2. If 38.25 ≤ *HWR* < 40.75 → Ectomorphy = 0.463 × *HWR* − 17.63.
3. If *HWR* < 38.25 → Ectomorphy = 0.1.

#### 2.5.3. Mesomorphy Calculation

The mesomorphic component, representing muscularity and skeletal robustness, was computed from standard anthropometric measurements available from the 3D scanner, using the Heath–Carter equation:

(5) Mesomorphy=0.858 ∗ breadth of humerus+0.601 ∗ breadth of femur+0.188 ∗ biceps circumference +0.161 ∗ calf circumference−height ∗ 0.131+4.5

where all measurements (breadths, circumferences, height) are expressed in centimeters (cm).

Here, the humerus and femur breadths, as well as limb circumferences, were extracted directly from the 3D scan data; these measurements were not adjusted based on skinfold thickness.

### 2.6. Data Evaluation

To evaluate the accuracy of the 3D scanner-derived somatotype estimates, the results were compared against manual measurements taken using the following:

- Skinfold calipers for reference endomorphy values.
- Measuring tape for reference circumferences.
- Bone calipers for reference skeletal breadths.

The accuracy of the 3D scan-based somatotype estimation was evaluated by comparing each participant’s predicted values with reference values obtained from manual anthropometry. Statistical agreement was assessed using Bland–Altman analysis, standard error of estimate (SEE), intraclass correlation coefficients (ICC), standardized measurement error (SME), coefficient of variation (CV), and root mean square error (RMSE). These metrics were computed for each somatotype component (endomorphy, mesomorphy, and ectomorphy) on the training dataset (n = 117).

To assess the generalizability of the model, an external validation was performed using an independent dataset consisting of 15 participants not included in the training dataset. The same procedures were used to acquire both manual and 3D scan data. The results were compared using Bland–Altman analysis, intraclass correlation coefficients (ICCs), standard error of estimate (SEE), root mean square error (RMSE), standardized measurement error (SME), and coefficient of variation (CV). The data analysis was performed using Microsoft Excel (Microsoft Corporation, USA) and GraphPad Prism 10 (GraphPad Software 10, Boston, MA, USA).

## 3. Results

Table 1 presents the demographic and anthropometric characteristics of the study participants (N = 117), consisting of 68 females and 49 males. The mean age of the sample was 22.1 ± 4.63 years with an average body weight of 69.86 ± 14.49 kg and an average height of 171 ± 8.98 cm.

The table further compares circumferences, limb breadths, and body segment dimensions obtained through manual measurement and 3D scanning. In most cases, 3D-derived measurements were slightly larger than manual values particularly for waist circumference (82.37 ± 11.03 cm vs. 80.85 ± 12.25 cm) and hip circumference (102.64 ± 8.44 cm vs. 98.64 ± 8.58 cm), suggesting a potential systematic overestimation by the 3D scanner.

Similarly, biceps circumference measured manually (30.54 ± 4.40 cm) was slightly lower than the 3D-derived value (31.56 ± 4.17 cm), whereas calf circumference showed minimal discrepancy (37.00 ± 3.46 cm manually vs. 36.75 ± 3.10 cm from 3D scanning), indicating better alignment between methods for certain body segments.

For bone breadths, the 3D-derived femur breadth (12.19 ± 1.05 cm) was notably larger than the manual measurement (10.30 ± 1.76 cm), whereas humerus breadth showed a smaller discrepancy (7.40 ± 0.85 cm in 3D vs. 7.18 ± 1.07 cm manually). These differences may arise from variations in anatomical landmark detection and measurement techniques between manual and 3D methods.

### 3.1. Body Height Prediction

Since the TC^2^ NX-16 full-body 3D scanner does not capture head height, height was predicted using the height of the *C7* vertebra based on a regression model developed from manually measured data:

(6) Predicted Height=a ∗ height C7 vertebra (mm)+b

where *a* (*a* = 0.1039) and *b* (*b* = 19.39) are regression coefficients determined from the study’s dataset.

**Figure 2:**
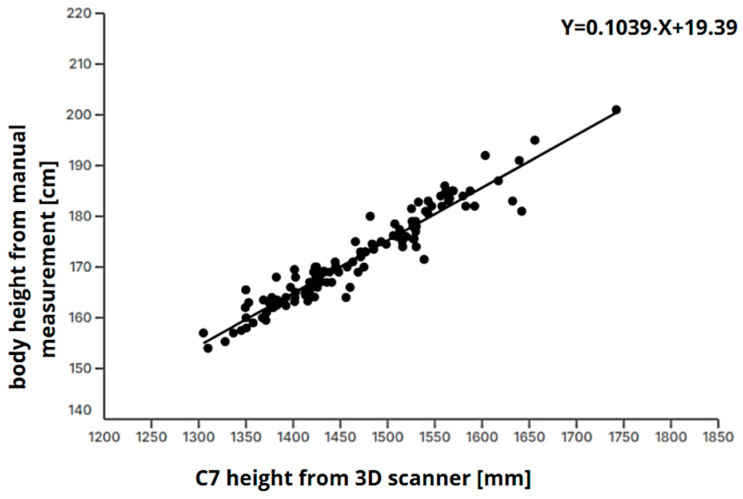
Scatter plot with a linear regression line representing relationship between body height and height of *C7* vertebra obtained from 3D body scanner.

This linear regression analysis (Figure 2) demonstrated a strong relationship between manually measured height (Y) and *C7* vertebra height obtained from a 3D full-body scanner (X). With a high coefficient of determination (R^2^ = 0.9218), it can be concluded that 92.18% of the variability in manually measured height is explained by the *C7* height from the scan, indicating a high accuracy of this method for predicting body height.

The regression equation

Y = 0.1039 ∗ X+ 19.39(7)

shows that manually measured height increases proportionally with *C7* vertebra height, where each increase of 1 unit in X corresponds, on average, to an increase of 0.1039 units in manually measured height. The slope value is statistically significant (*p* < 0.0001), confirming that *C7* height is a reliable predictor of total body height.

The low standard error of estimation (Sy.x = 2.535) indicates that the model’s predictions are accurate and stable, while the confidence interval for the slope (0.09840 to 0.1094) suggests that this relationship remains consistent across different samples.

Since this predictive equation for estimating body height from 3D scans has demonstrated high accuracy, it was used for the automatic estimation of the ectomorphic component to ensure consistency in body composition assessment. In 117 subjects, the average difference between actual height and predicted height was 1.83 cm ± 1.72 (SD).

### 3.2. Endomorphy Evaluation

Table 2 compares the endomorphy values obtained using two different methods—the 3D method (Endomorphy 3D) and the traditional method (Endomorphy).

The average endomorphy value obtained through 3D scanning was 5.10 ± 1.51, while the traditional method yielded 4.80 ± 1.58. The difference between these methods averaged 1.33 ± 1.19.

### 3.3. Ectomorphy Evaluation

The results show a high agreement between the two methods with a minimal difference of 0.27 ± 0.30 (Table 3). Similar standard deviations indicate that both methods capture a comparable range of variability within the sample.

### 3.4. Mesomorphy Evaluation

The fully 3D-derived mesomorphy values (Table 4) were higher (7.31 ± 2.24) compared to manual measurement (6.04 ± 2.58), suggesting that 3D scanning may slightly overestimate mesomorphy. However, when breadths were manually measured and incorporated into the 3D model, the values (5.98 ± 2.53) closely matched manual measurements. The small average difference (0.27 ± 0.30) suggests that 3D scanning is a reliable method for assessing body composition despite minor systematic variations.

### 3.5. Somatotype Determination Analysis

Overall, the agreement between manually determined and 3D-classified somatotypes, based on the 13-type classification system derived from W.H. Sheldon’s broader somatotype categorization, reached 87.18% with a 12.82% discrepancy. The most frequent differences occurred in the endomorph category (5 cases), which was followed by the mesomorph (3 cases) and ectomorph (3 cases) categories. Minor deviations were also observed in mesomorph–endomorph (2 cases), central type (1 case), and ectomorph–endomorph (1 case). The results demonstrate moderate to high concordance in most cases with some limitations in endomorph classification.

### 3.6. Data Analysis

The analysis comparing body composition components obtained through manual measurement and 3D scanning (Table 5) demonstrated a high level of agreement across all three somatotype components (endomorphy, mesomorphy, and ectomorphy).

The statistical analysis demonstrated a high degree of agreement and reliability between 3D scanning and manual measurement methods across all three somatotype components (ectomorphy, mesomorphy, and endomorphy). Mean differences were minimal (0.02 for ectomorphy and mesomorphy, 0.25 for endomorphy), indicating that 3D scanning provides highly comparable results to traditional manual techniques. The limits of agreement (LoA) remained within acceptable ranges, confirming that the variability between the two methods is low. Additionally, the high intraclass correlation coefficients (ICC ≥ 0.87) further support the strong consistency and reproducibility of 3D-derived values. The low standard error of estimate (SEE), root mean square error (RMSE), and standard measurement error (SME) reinforce the precision of 3D scanning, particularly for ectomorphy and mesomorphy, where the differences were negligible. Endomorphy exhibited a slightly higher discrepancy, suggesting a potential overestimation of body fat by 3D scanning, which was likely due to differences in the way soft tissue is accounted for compared to traditional skinfold measurements. The evenly distributed differences across measurement ranges further support the absence of systematic bias, reinforcing the reliability of 3D scanning for automatic and efficient body composition assessment.

### 3.7. External Validation Using Independent Test Sample

To further assess the generalizability of the proposed model, an external validation was conducted (Table 6) on an independent sample of 15 individuals who were not involved in the model development phase.

Table 7 presents a summary of statistical indicators comparing the 3D-derived and manually measured somatotype components based on an external and independent validation group.

The results demonstrated high agreement between manual and 3D-derived values across all three somatotype components. Ectomorphy showed excellent concordance (RMSE = 0.35; ICC = 0.97), which was followed by mesomorphy (RMSE = 0.82; ICC = 0.86) and endomorphy (RMSE = 1.10; ICC = 0.85). The narrow limits of agreement and low measurement errors further support the reliability of the 3D-based method in capturing individual somatotype profiles.

**Figure 3:**
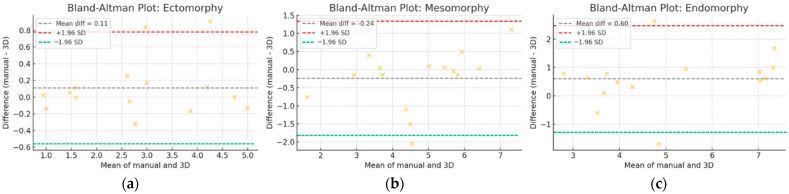
Bland-Altman plots for (**a**) ectomorphy, (**b**) mesomorphy, and (**c**) endomorphy, where yellow crosses represent individual differences between manual and 3D measurements versus their mean, with dashed lines indicating the mean difference (gray), the upper limit of agreement +1.96 SD (red), and the lower limit of agreement −1.96 SD (green).

Bland–Altman analysis (Figure 3) was performed to visually assess the agreement between manual and 3D-derived somatotype components. The plots revealed a small positive bias for endomorphy (mean difference = + 0.60) and a near-zero bias for ectomorphy (+0.11), indicating good overall agreement. However, mesomorphy showed a noticeable negative bias (−1.93), suggesting that the 3D scanner may overestimate muscularity due to differences in how breadth dimensions are extracted.

## 4. Discussion

The findings of this study demonstrate that full-body 3D scanning provides a viable and reliable alternative to traditional manual somatotyping methods. Although 3D-derived somatotype components showed general alignment with manual measurements, notable discrepancies—especially in endomorphy and mesomorphy—underscore limitations stemming from methodological differences in data acquisition and calculation procedures.

One notable finding is the systematic overestimation of body circumferences by the 3D scanner compared to manual measurements. This tendency was most evident in waist and hip circumferences, suggesting that the scanner may account for body surface irregularities differently than manual tape measurements. This discrepancy contributed to the slight overestimation of endomorphy values, as endomorphy is heavily dependent on body fat estimation, which in this study was derived using the U.S. Navy body fat equation rather than direct skinfold thickness measurements. Despite this limitation, the 3D-derived endomorphy values remained within acceptable limits when compared to manual assessments [4,12,14,15].

Regarding ectomorphy, the minimal differences between 3D-derived and manual measurements highlight the robustness of the height-to-weight ratio (HWR) method for predicting linearity and leanness. The strong correlation between manually measured height and the predicted height from the *C7* vertebra height further supports the accuracy of the regression-based height estimation method employed in this study.

Mesomorphy calculations presented a more complex picture. The initial 3D-derived mesomorphy values were slightly higher than manual measurements, which was likely due to subtle differences in bone breadth measurements. However, when bone breadths were manually measured and incorporated into the 3D model, the results closely matched the manual reference values. This finding suggests that 3D scanning may be particularly sensitive to variations in anatomical landmark detection, which could introduce minor inconsistencies in skeletal breadth assessments. Nonetheless, the overall agreement in mesomorphy measurements confirms that 3D scanning can approximate skeletal robustness, though certain anatomical landmarks remain prone to overestimation [16,17].

One important aspect is the standard deviation (SD), which represents the variability of values within the studied sample. In this case, the SD values for both methods are very similar (min. difference ± 0.03, max. difference ± 0.29), indicating that the dispersion of values is almost identical. This fact suggests that both methods can capture the same level of variability among individuals—that is, some individuals have higher endomorphy values, others lower, and this variability remains unchanged regardless of the method used.

Since standard deviation reflects natural differences between people (for example, some individuals may be more endomorphic, while others less so), its similarity between methods is a positive indicator of measurement consistency. If the SD of the 3D method was significantly higher or lower than that of the traditional method, it could indicate that one method is not sufficiently accurate or has difficulty precisely capturing body variation within the population [4,18].

The statistical analysis further reinforces the reliability of 3D scanning for somatotyping. The high intraclass correlation coefficients (ICC ≥ 0.87) indicate strong consistency between the two measurement techniques. The low standard error of estimate (SEE), root mean square error (RMSE), and standard measurement error (SME) demonstrate the precision of 3D scanning particularly for ectomorphy and mesomorphy. While endomorphy exhibited slightly higher variability, the Bland–Altman plots confirmed that the overall agreement between the two methods remained within acceptable ranges with no significant systematic bias.

In the external validation on an independent sample (n = 15), this trend persisted—ectomorphy achieved excellent agreement (ICC = 0.97, RMSE = 0.35), whereas mesomorphy (ICC = 0.86) and especially endomorphy (ICC = 0.85, RMSE = 1.10) showed higher variability. Elevated values of standard error and coefficient of variation for endomorphy (CV = 17.78%) suggest potential systematic deviations particularly for individuals with extreme body types. Although the limits of agreement for endomorphy were relatively wider, the overall results suggest that the method generalizes well to unseen data. However, due to the limited size of the validation sample, these findings should be interpreted with caution and confirmed in future studies with larger and more diverse populations. For these reasons, 3D scanning should currently be considered a promising supplementary tool for somatotype estimation—with very good accuracy for ectomorphy and acceptable performance for mesomorphy but limited reliability for endomorphy. The latter requires further methodological refinement and validation before it can be considered a standalone replacement for traditional anthropometric techniques.

Given these results, the integration of 3D scanning technology in somatotyping offers several advantages. First, it provides a rapid, non-invasive, and standardized method for body composition assessment, reducing the reliance on trained professionals and minimizing inter-observer variability. Second, automated measurements ensure greater reproducibility particularly for ectomorphic and mesomorphic classifications. Finally, the ability to integrate predictive models for body height and body fat estimation further enhances the versatility of 3D scanning in anthropometric research and practical applications in sports science, medicine, and health monitoring [2,19].

## 5. Conclusions

As a partial replacement of manual somatotyping, 3D scanning shows promising potential particularly for ectomorphy and mesomorphy, while endomorphy estimates still require refinement. The results indicate strong agreement between 3D-derived and manual measurements for ectomorphy, mesomorphy, and endomorphy with minor discrepancies largely attributable to differences in measurement techniques. The 3D scanning approach offers several benefits, including enhanced accuracy, standardization, and efficiency, making it a valuable tool for body composition assessment in various fields.

Despite its advantages, 3D scanning still presents some limitations, particularly in the estimation of endomorphy, where direct skinfold measurements remain the gold standard. Future research should focus on refining predictive models for body fat estimation and improving anatomical landmark detection algorithms to enhance measurement accuracy further. Additionally, integrating machine learning techniques could improve the automated classification of somatotypes, making the process even more precise and accessible.

Although the external test sample included only 15 individuals, the observed consistency and low error rates offer meaningful preliminary validation. Future studies should aim to expand the validation sample to confirm generalizability across broader populations. It is important to expand the sample size and include individuals with extreme somatotype characteristics in order to better evaluate the model’s applicability across diverse body types. Overall, the findings highlight the potential of 3D scanning as a transformative technology in anthropometric studies, sports science, and medical diagnostics.

## Figures and Tables

**Table 1:** Participant characteristics and anthropometric measurements.

N = 117	Average ± SD
Gender	68 females, 49 males
Age (years)	22.1 ± 4.63
Weight (kg)	69.86 ± 14. 49
Height (cm)	171 ± 8.98
Waist circumference (cm)	80.85 ±12.25
Waist circumference 3D (cm)	82.37 ± 11.03
Hips circumference (cm)	98.64 ± 8.58
Hips circumference 3D (cm)	102.64 ± 8.44
Biceps circumference (cm)	30.54 ± 4.40
Biceps circumference 3D (cm)	31.56 ± 4.17
Calf circumference (cm)	37.00 ± 3.46
Calf circumference 3D (cm)	36.75 ± 3.10
Humerus breadth (cm)	7.18 ± 1.07
Humerus breadth 3D (cm)	7.4 ± 0.85
Femur breadth (cm)	10.30 ± 1.76
Femur breadth 3D (cm)	12.19 ± 1.05

**Table 2:** Comparison of the average endomorphic component values obtained through 3D scanning and manual measurement.

N = 117		Average ± SD
Endomorphy 3D		5.10 ± 1.51
	Female	5.88 ± 1.04
	Male	4.31 ± 1.49
Endomorphy		4.80 ± 1.58
	Female	5.21 ± 1.58
	Male	4.40 ± 1.62
Overall difference		1.33 ± 1.19

**Table 3:** Comparison of the average ectomorphic component values obtained through 3D scanning and manual measurement.

N = 117		Average ± SD
Ectomorphy 3D		2.30 ± 1.48
	Female	2.63 ± 1.45
	Male	1.98 ± 1.52
Ectomorphy		2.31 ± 1.51
	Female	2.65 ± 1.37
	Male	1.96 ± 1.64
Difference		0.27 ± 0.30

**Table 4:** Comparison of the average mesomorphic component values obtained through 3D scanning and manual measurement.

N = 117		Average ± SD
Mesomorphy 3D		7.31 ± 2.24
	Female	6.69 ± 2.39
	Male	7.93 ± 2.09
Mesomorphy 3D (breadths measured manually)		5.98 ± 2.53
	Female	5.84 ± 2.62
	Male	6.18 ± 2.38
Mesomorphy		6.01 ± 2.59
	Female	5.88 ± 2.76
	Male	6.19 ± 2.34
Difference Mesomorphy vs. Mesomorphy 3D (breadths measured manually)		0.27 ± 0.30

**Table 5:** Results of the internal evaluation test which compared the manual measurement and the prediction from 3D scan on the training dataset (n = 117).

N = 117	M ± SD	LoA	SEE	ICC-a	SME-a	CV-a	RMSE	ICC-r	SME-r	CV-r
Ectomorphy	0.02 ± 2.24	−0.77, 0.88	2.53	0.92	0.12	3.1%	0.15	0.91	0.12	2.90%
Mesomorphy	0.02 ± 2.53	−1.24, 1.27	2.24	0.91	0.14	2.8%	0.18	0.90	0.14	2.60%
Endomorphy	0.25 ± 2.53	−3.25, 3.75	1.72	0.89	0.19	3.6%	0.22	0.87	0.19	3.30%

Mean ± SD, mean difference, limits of agreement: 95% limit of agreement (LoA), standard error of estimate (SEE), intraclass correlation (ICC), coefficient of variation (CV), root mean square error (RMSE), and standard measurement error (SME).

**Table 6:** Characteristics of the external validation test sample and their anthropometric measurements.

N = 15	Average ± SD
Gender	9 females, 6 males
Age (years)	20.8 ± 1.1
Weight (kg)	64.50 ± 9. 12
Height (cm)	171.8 ± 9.33
Waist circumference (cm)	72.40 ± 7.14
Waist circumference 3D (cm)	74.84 ± 7.38
Hips circumference (cm)	94.00 ± 6.47
Hips circumference 3D (cm)	99.54 ± 6.14
Biceps circumference (cm)	26.83 ± 3.12
Biceps circumference 3D (cm)	29.70 ± 2.93
Calf circumference (cm)	36.30 ± 2.05
Calf circumference 3D (cm)	36.83 ± 3.00
Humerus breadth (cm)	7.00 ± 5.32
Humerus breadth 3D (cm)	7.00 ± 0.85
Femur breadth (cm)	9.40 ± 0.89
Femur breadth 3D (cm)	12.26 ± 1.30

**Table 7:** Results of the external evaluation test which compared the manual measurement and the prediction from 3D scan on the independent validation sample (n = 15).

N = 15	M ± SD	LoA	SEE	SME	CV	RMSE	ICC
Ectomorphy	0.11 ± 0.34	−0.56, 0.78	1.82	0.09	11.83%	0.35	0.97
Mesomorphy	−0.24 ± 0.81	−1.53, 1.34	10.01	0.21	17.67%	0.82	0.86
Endomorphy	0.60 ± 0.96	−1.27, 2.48	18.24	0.25	17.78%	1.10	0.85

Mean ± SD, mean difference, limits of agreement: 95% limit of agreement (LoA), standard error of estimate (SEE), intraclass correlation (ICC), coefficient of variation (CV), root mean square error (RMSE), and standard measurement error (SME).

## Data Availability

The original contributions presented in the study are included in the article, further inquiries can be directed to the corresponding author.
